# Flexible pseudotyping of retrovirus using recombinase-mediated cassette exchange

**DOI:** 10.1007/s10529-018-2515-6

**Published:** 2018-01-20

**Authors:** Hugo R. Soares, Ana I. Almeida, Hélio A. Tomás, Paula M. Alves, Ana S. Coroadinha

**Affiliations:** 1 iBET – Instituto de Biologia Experimental e Tecnológica, Apartado 12, 2781-901 Oeiras, Portugal; 2 Instituto de Tecnologia Química e Biológica António Xavier, Universidade Nova de Lisboa, Av. da República, 2780-157 Oeiras, Portugal

**Keywords:** Amphotropic envelope, GalLV envelope, Pseudotyping, Recombinase, Retrovirus, RMCE, VSV-G envelope

## Abstract

**Objective:**

Develop an engineered cell line containing two flexible gene expression systems enabling the continuous production of tailor-made recombinant gammaretrovirus with predictable productivities through targeted integration.

**Results:**

Dual-FLEX cells (dFLEX) contain two independent recombinase-mediated cassette exchange (RMCE) systems which confer flexibility to the expression of different transgene and envelope combinations. The flexible envelope expression in dFLEX cells was validated by pseudotyping retrovirus particles with three different viral envelope proteins—GaLV, 4070A and VSV-G. Our results show that dFLEX cells are able to provide high titers of infectious retroviral particles with a single-copy integration of the envelope constructs after RMCE. The integrated CRE/*Lox* tagging cassette was amenable to express envelope proteins both using constitutive (i.e. CMV) and inducible (i.e. Tet-on) promoters.

**Conclusions:**

dFLEX cell line provides predictable productivities of recombinant retrovirus pseudotyped with different envelope proteins broadening the tropism of particles that can be generated and thus accelerating the research and development of retrovirus-based products.

**Electronic supplementary material:**

The online version of this article (10.1007/s10529-018-2515-6) contains supplementary material, which is available to authorized users.

## Introduction

Murine leukemia viruses (MLV)-derived viral vectors are the core of several research tools widely used in biotechnology; their most attractive features are (i) the ability to integrate the viral genome into hosts’ genome (Debyser et al. 2015), (ii) the flexible tropism (Maetzig et al. 2011) (iii) the low immunogenicity (Soares et al. 2016) (iv) and, no cellular toxicity allowing stable and continuous particle production (Miller 1990). The wide use of retroviral vectors was initially hindered due to the relatively low titers of infectious particles attained (Cone and Mulligan 1984; Danos and Mulligan 1988). A variety of parameters such as (i) the origin of the cell line (Rodrigues et al. 2013), (ii) promoter strength and competition (Schambach et al. 2006) (iii) and culturing conditions (Coroadinha et al. 2006a, b, d) can affect vector productivity. To minimize these impacts and to accelerate the manufacturing platform packaging cell lines were designed. More recently, modular packaging cell lines such as Flp293A (Schucht et al. 2006) and 293FLEX (Coroadinha et al. 2006c) introduced the advantage of flexible expression of different transgenes in pre-defined high-expression chromosomal integration site(s). These cells were specifically designed to produce viral vectors with defined tropisms. However, the rapid evolving field of gene therapy is now asking for vectors with increased tissue specificities as a mechanism to direct target gene delivery into a specific tissue. In this work, we report the introduction of a second modular expression cassette into dFLEX cells offering flexibility to the envelope used for vector pseudotyping. This cassette is flanked by Cre/*Lox* recombination sites and works independently from the previously integrated retroviral genomic transgene expression cassette which is flanked by Flp/*FRT* recombination sites. The newly engineered cell line, named dual-FLEX (dFLEX) by holding two independent RMCE systems, allows tailoring and fasten the production of recombinant retroviral vectors with different combinations of envelope and vector transgene expression cassettes.

## Materials and methods

### Cell line development: transfection, selection, and cloning

To tag 293#3 gp11 and 293#3 gp22 cell lines (Coroadinha et al. 2006c; Carrondo et al. 2008) with the Cre/*Lox* cassette for site-specific recombination, cells were electroporated with pTagLoxPGFP-zeo using the NeoTransfection system (Thermo Fisher Scientific) according to manufacturer’s instructions (0.5 µg DNA per 10^6^cells 1200 V, 20 ms, 2 pulses). Two days later, antibiotic selection was initiated by replacing culture medium with fresh DMEM (Gibco) supplemented with 10% (v/v) FBS, 200 μg mL^−1^ of Zeocin (Invivogen). eGFP-positive cell cloning was performed by MoFlo high-speed cell sorter (Beckman Coulter), 30 clones with medium and 30 clones with high fluorescence were collected and expanded in 50% (v/v) conditioned medium, 20% (v/v) FBS and 200 μg mL^−1^ of Zeocin. Surviving clones with high fluorescence were analyzed for single-copy cassette integration by RT-qPCR using as single-copy reference 293 FLEX-eGFP clone 293#3 gp22 Env 8 (Coroadinha et al. 2006c). To determine the percentage and intensity of eGFP-positive cells, a CyFlow Space (Sysmex, Horgen, Switzerland) flow cytometer was used. For further details on the cell lines, culture conditions and plasmid cloning please consult the Supplementary Information (‘Supplementary Materials and Methods’).

### Recombinase mediated cassette exchange

For Cre/*Lox* site-specific cassette exchange, dFLEX#11 and dFLEX#22 cells were seeded at 5 × 10^4^ cells per cm^2^ in six-well plates and co-transfected the next day with 2 μg of targeting envelope plasmid and 6 μg of pZeoCre recombinase-expressing plasmid per 10^6^ cells, after mixing with PEI in a ratio of 1 μg of DNA to 1.5 μg of PEI. After 21 days under selective pressure, puromyci- resistant, eGFP-negative, surviving cells were isolated and cloned by limiting dilution in 50% conditioned media, 20% (v/v) FBS and 15 µg ml^−1^ of puromycin, amplified and cryopreserved before further analysis. Details on the PCR and RT-PCR to characterize the cells are indicated as ‘Supplementary Materials and Methods’.

### Titration of infectious viral particles

Production and titration of infectious retroviral particles pseudotyped with gibbon ape leukemia virus (GaLV), amphotropic murine leukemia virus envelope (4070A) and vesicular stomatitis virus glycoprotein (VSV-G) were performed as described in Coroadinha et al. (2006c). Briefly, RD cells were seeded at 5 × 10^4^ cells cm^−2^ in 96-well plates 1 day before infection. Transduction was performed in triplicates by removing the cell supernatant and infecting with 0.05 mL of filtered viral suspension supplemented with 8 mg mL^−1^ of polybrene (Sigma-Aldrich). Cells were incubated at 37 °C for 4 h after which 0.2 mL of fresh medium was added. Two days after infection, cells were fixed, washed and stained. The viral titer was determined by counting the stained blue cells using a phase-contrast inverted microscope. Further details on infectious particles production are indicated as Supplementary Information (‘Supplementary Materials and Methods’).

## Results

### Development of a dual-FLEX cell line for versatile production of retroviral vectors

**Fig. 1:**
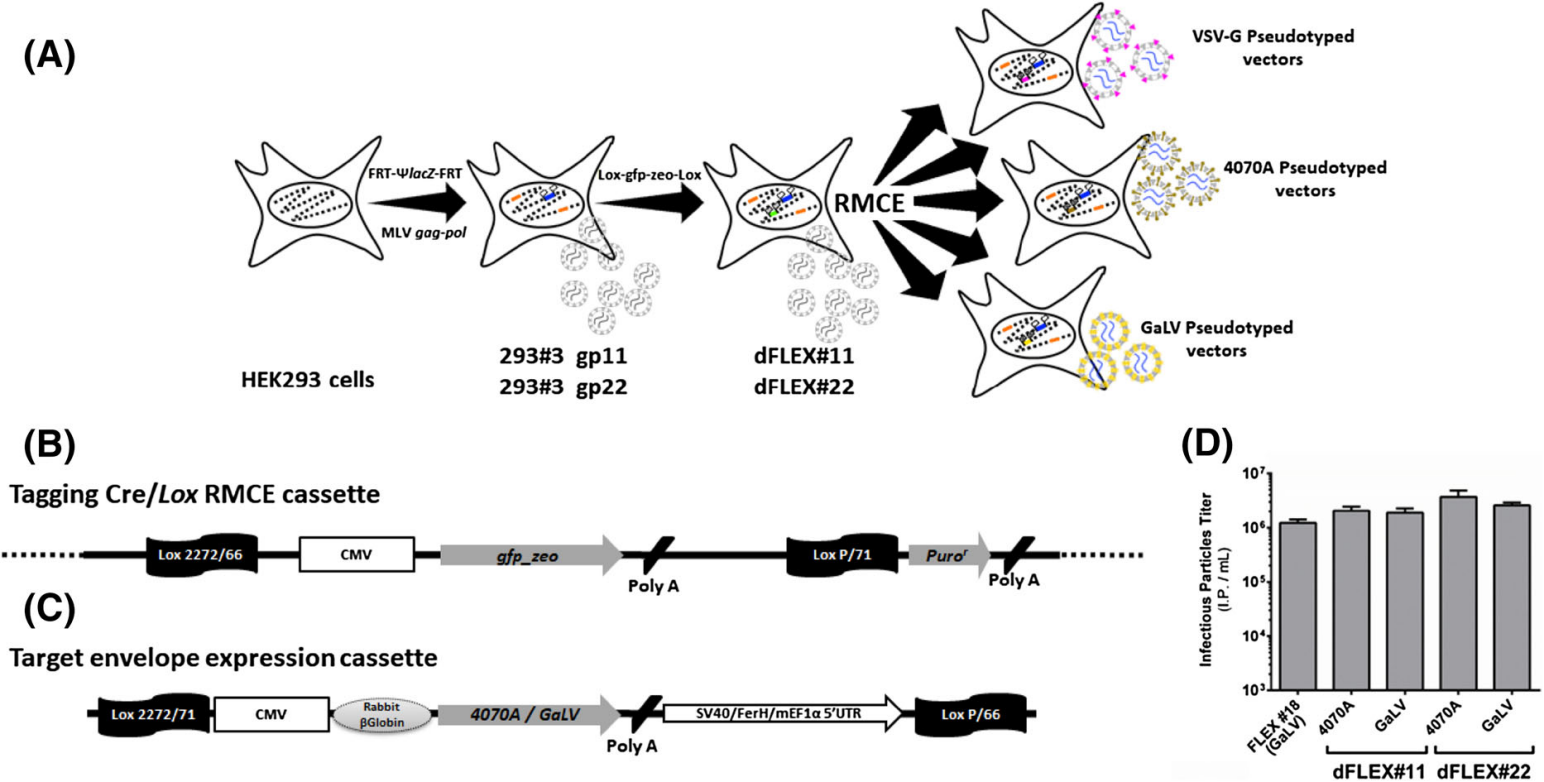
Generation of the recombinant retrovirus producer cell line with exchangeable transgene and envelope expression cassettes. **a** Schematic representation of cell line development strategy, HEK293-derived cells stably transfected with MLV gag-pol gene and tagged with a Flp/*FRT* RMCE cassette for flexible transgene expression (Coroadinha et al. 2006c), were tagged with a Cre/*Lox* RMCE cassette for flexible envelope expression. Orange-filled boxes represent *gag*-*pol* introduced with random integration; Blue and Green colored boxes with small white flags represent both RMCE systems introduced for flexible transgene and envelope exchange; Pink, brown and yellow colored boxes with crossed flags represent envelope expression cassettes after RMCE. **b** Schematic representation of pTagLoxPGFP-zeo plasmid containing a CMV promoter, a GFP-zeo reporter gene, two polyadenylation sequences, two Lox sites and a promoterless puromycin resistance gene. **c** Schematic representation of the targeting plasmid containing, CRE/*Lox* recombination sites, a CMV promoter, Rabbit beta-globin intron, the envelope of interest, a polyadenylation sequence and a composite promoter (SV40/FerH/mEF1α5’UTR) to drive puromycin expression after successful RMCE. **d** Production titers determined for pseudotyped retrovirus produced after RMCE. FLEX #18 is a control cell line where GaLV envelope was introduced by random plasmid integration (Coroadinha et al. 2006c). Error bars correspond to standard deviations (*n* ≥ 3)

To generate a cell line for the versatile production of MLV-based retroviral products with tailored retroviral genome and envelope, 293#3 gp11 and 293#3 gp22 cells previously developed containing one Flp/*FRT* RMCE system were engineered to insert a second RMCE system based on Cre/*Lox* (Fig. 1a). Both 293#3 gp11 and 293#3 gp22 cell lines were electroporated with pTagLoxPGFP_zeo tagging plasmid (Fig. 1b). Electroporation efficiency—measured 1 week after electroporation and determined by the percentage of eGFP-positive cells in unselected populations—was between 68 and 70% for both 293#3 gp11 and 293#3 gp22 cell lines. After 21 days of zeocin selection, resistant cells were sorted into single-cell clones by flow cytometry. Of these, 18 high-eGFP expression clones derived from each parental cell line were analyzed for single-copy integration by RT-qPCR. Our data indicated that 17/18 (94%) of 293#3 gp11-derived clones and 15/18 (84%) of 293#3 gp22-derived clones contained only one single copy of the tagging plasmid. One clone of each tagging cell line was selected for further studies and named dFLEX#11 and dFLEX#22, respectively.

### Production of GaLV and 4070A pseudotyped retrovirus particles using Cre/*Lox* RMCE

Both dFLEX#11 and dFLEX#22 cell lines were subjected to Cre/*Lox* RMCE using two different targeting plasmids for the constitutive expression of (i) 4070A or (ii) GaLV viral envelopes (Fig. 1c). The overall RMCE efficiency was approximately 2%, determined by the percentage of eGFP-negative cells in unselected population 48 h after co-transfection. After enriching the population for eGFP-negative cells with puromycin selection, surviving cells were cloned by limiting dilution. As observed in Fig. 1d, all pseudotypes generated infectious particles titers above 10^6^ IP mL^−1^. In this study, FLEX#18 cell line was used as reference; FLEX#18 derives from 293#3 gp22 in which GaLV envelope was introduced using classical transfection and random integration method. The overall titers of both dFLEX#11 and dFLEX#22-derived clones were in average 1.5-fold to threefold higher than those of related FLEX#18 cells suggesting that a single copy of the envelope cassette is sufficient to provide high titers of infectious retroviral vectors.

### Characterization of dFLEX cells after RMCE

**Fig. 2:**
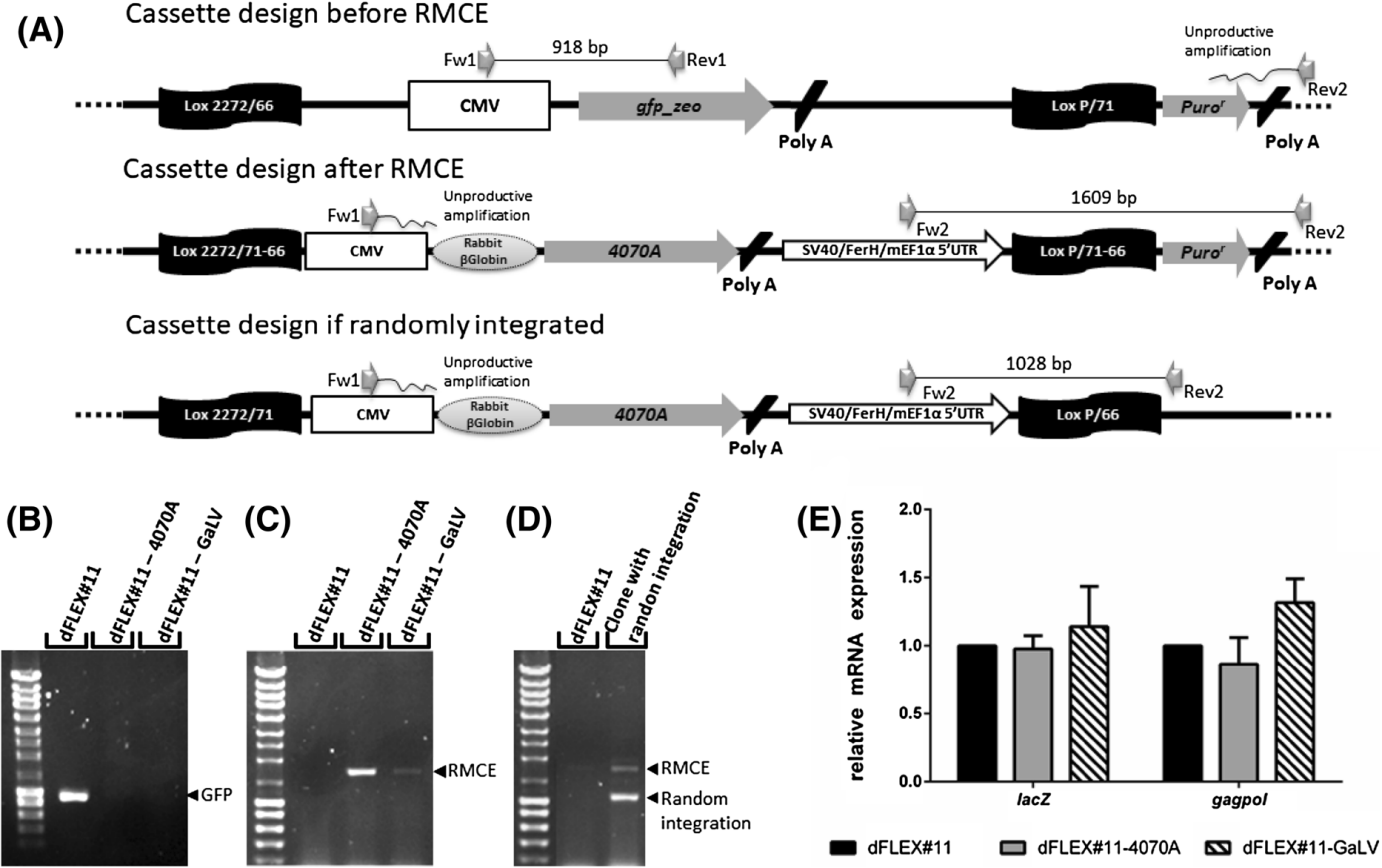
Characterization of targeted clones. **a** Schematic representation of the PCR design to confirm correct cassette exchange and analysis of potential random integration of the targeting plasmid. The pair of primers (gray arrows) used to discriminate targeted from random integration of the tagging plasmid are shown. **b** Example of PCR amplification products obtained for parental dFLEX cells, two correctly targeted clones and a cell clone with additional random cassette integration using the primer set. **b** Fw1 + Rev1 and **c** and **d** Fw2 + Rev2; **e** Relative mRNA expression levels of *lacZ* and MLV *gag-pol* genes in dFLEX#11—derived clones, 0.5-fold to 1.5-fold are considered as lower and top limits of no-change in relative mRNA expression. Error bars correspond to standard deviations (*n* ≥ 3)

RMCE efficiency was evaluated by the occurrence of both site-specific integration, with the removal of the tagging reporter gene, and additional random integration of the expression plasmid in the genomic DNA. The efficient removal of the eGFP-containing tagging cassette was evaluated using a set of primers binding to the CMV promoter and to the eGFP reporter gene (Fig. 2a). The amplification of a 918-bp PCR product associated to the tagging cassette was detected only in parental cell lines but not in any of the 28 individual clones screened (Fig. 2b) suggesting 100% RMCE efficiency. In addition, the discrimination between RMCE-mediated targeted integration and additional random integration events was performed using standard PCR with a specifc set of primers recognizing regions upstream and downstream of the LoxP/66 recombination site (Fig. 2a). Our data confirmed that 26/28 (93%) clones analyzed were correctly exchanged showing amplification of a single 1609-bp PCR product (Fig. 2c) while in the two other clones (7%) it was possible to observe an additional 1028-bp PCR amplification product (Fig. 2d) suggesting random integration occurring in parallel with RMCE-mediated integration. In addition, to evaluate if RMCE and subsequent cloning steps had any impact in the expression of MLV *lacZ* and *gag-pol* expression, the relative mRNA expression levels of these genes in the selected clones were compared to the parental dFLEX cells. No major differences in the expression of neither *lacZ* nor *gag-pol* were detected (Fig. 2e), hence suggesting no impact in the expression of the recombinant retrovirus genome and structural genes.

### Expression of VSV-G in dFLEX cells using Cre/*Lox* RMCE

**Fig. 3:**
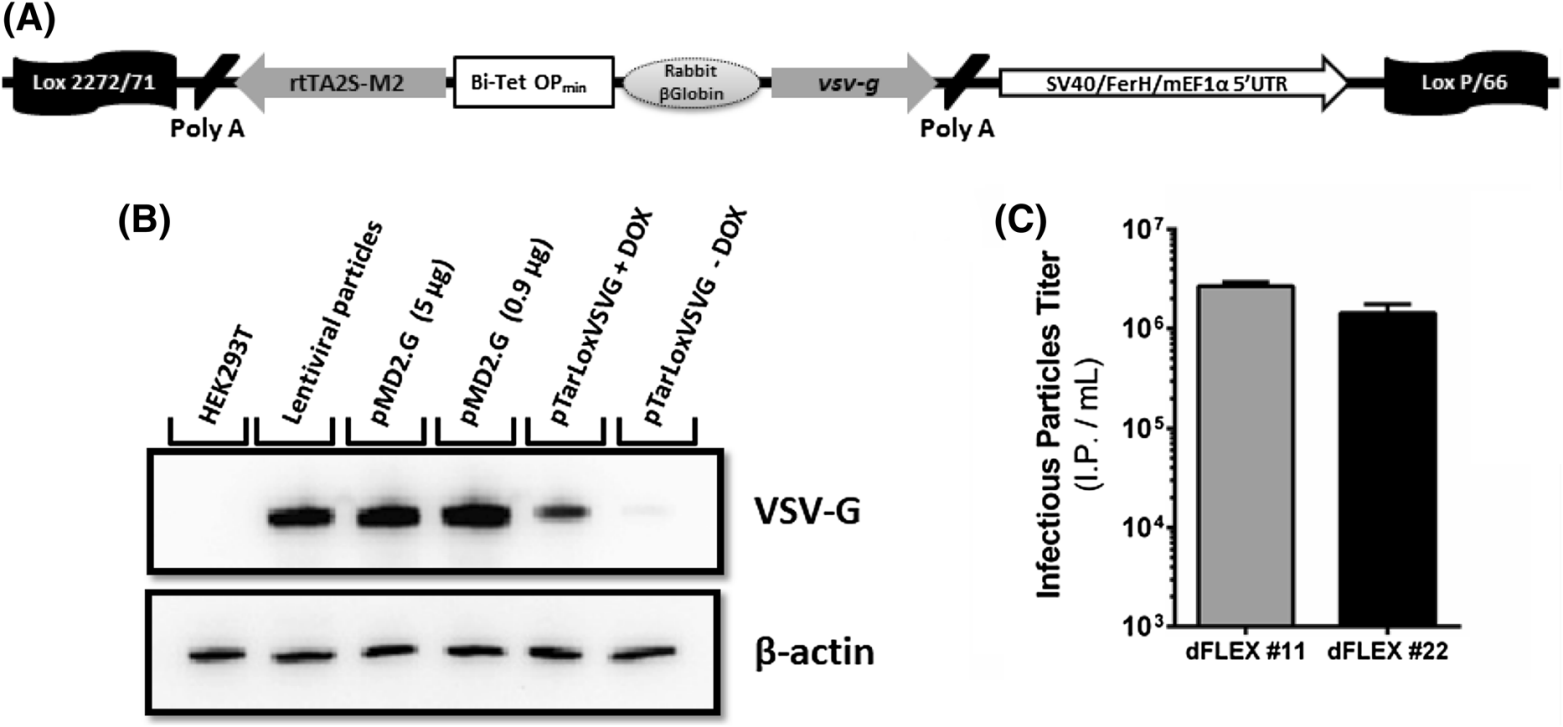
Inducible expression of VSV-G envelope proteins. **a** Schematic representation of the targeting plasmid containing, CRE/*Lox* recombination sites, a bidirectional promoter responsive to tetracycline, a Rabbit beta-globin intron, VSV-G envelope gene, two polyadenylation sequences and a composite promoter (SV40/FerH/mEF1α5’UTR) to drive puromycin expression after successful RMCE. **b** Western blotting showing the effective expression of VSV-G protein after doxycycline induction; HEK293T cell extracts were used as negative control, three positive controls were used, second lane (i) an extract of HEK293T cells producing VSV-G pseudotyped lentiviral particles and third and fourth lanes cellular extracts of HEK293T transiently transfected with VGV-G coding pMD2.G plasmid at (ii) 5 µg of pMD2.G or (iii) 0.9 µg pMD2.G + 4.1 stuffer DNA per 10^6^ cells; test samples in the fifth and sixth lanes are cellular extracts of HEK293T transiently transfected with 5 µg of pTarLoxVSVG plasmid per 10^6^ cells with and without doxycycline stimulus; β-actin staining indicates equivalent loading of all samples. **c** Volumetric titers of VSV-G pseudotyped retrovirus produced after inducing dFLEX-VSV-G cells with 2 µg ml^−1^ doxycycline for 24 h. Error bars correspond to standard deviations (*n* ≥ 3)

Having validated dFLEX cells for the production of pseudotyped recombinant retrovirus with predictable viral titers, dFLEX cells were challenged to express VSV-G envelope protein. Given the cytotoxicity associated with the constitutive expression of VSV-G, an inducible promoter responsive to tetracycline (Fig. 3a) was introduced to regulate gene expression levels. The functionality of this construct was assessed by determining the transient expression of VSV-G protein in cellular extracts 24 h after induction with doxycycline. As shown in Fig. 3b, VSV-G protein is detected in cells transfected with pTarLoxVSVG plasmid only after induction with doxycycline. The validated construct was then used for VSV-G expression in dFLEX cells after successful RMCE. As shown in Fig. 3c, the titer of infectious particles produced by dFLEX-VSV-G cells determined after doxycycline induction (2 µg ml^−1^, 24 h) was above 10^6^ IP mL^−1^. These results suggest the efficient pseudotyping of retrovirus particles with VSV-G envelope protein which validates the use of dFLEX cells for the production of recombinant retrovirus pseudotyped with toxic envelope proteins.

## Discussion

Recombinant retroviruses have been widely used to generate a variety of gene therapy candidates, vaccines candidates and research tools to generate induced pluripotent stem cells. In this work, the successful development of a new engineered cell line enables the production of a variety of recombinant retrovirus particles using two independent RMCE systems (Fig. 1a). In the literature, a limited number of multiplex cell lines combining different RMCE systems are described (Kameyama et al. 2010; Turan et al. 2010, 2014; Anderson et al. 2012), however, none couples the advantages of multiplexing to the tailored production of viral vectors. In this work, the transgene expression flexibility reported and validated for 293 FLEX (Coroadinha et al. 2006c) was extended with the flexibility of viral envelope(s) expression to produce tailor-made recombinant retrovirus.

The Flp/*FRT* system present in 293#3 gp11 and 293#3 gp22 cells (Coroadinha et al. 2006c; Carrondo et al. 2008) was introduced into the genome of parental cells by retrovirus transduction resulting in the duplication of FW and F5 sites which now impose two major limitations for multiplexing (i) a limited number of additional *FRT* mutant sequences that can ensure totally independent recombination, and (ii) the possible excision of the transgene expression cassette while performing FLP-based RMCE (Turan et al. 2010, 2013). As a result, the aimed flexibility in envelope expression was herein achieved with the introduction of a RMCE based on Cre/*Lox*P recombination system (Fig. 1a and Fig. 1b).

The successful integration of the tagging plasmid in producer cells’ genome occurred in more than 65% of unselected cells. Moreover, in more than 90% of high-expression clones only one copy of the tagging plasmid was incorporated. This single-copy integration is essential to enable the use of RMCE technology (Turan et al. 2011). To evaluate the flexibility of the Cre/*Lox*P RMCE system to express different viral envelopes 4070A or GaLV pseudotyped retrovirus were produced (Fig. 1c). Even though the RMCE efficiency determined in unselected cell population was near 2%, the entrapment of cells that undergone recombination through puromycin selection guaranteed 100% recombinant cells in the final population. All isolated clones analyzed were positive for cassette exchange although a small percentage also incorporated additional copies of the targeting cassette due to random integration events (Fig. 2d). Different individual cell clones produced up to threefold more infectious particles (Fig. 1d) than 293 FLEX, suggesting that one single copy of the gene encoding the envelope protein is sufficient to support high titers of retroviral particles. Both MLV genome and structural genes expression levels were maintained when compared with the parental cell line (Fig. 2e) suggesting that neither RMCE nor associated cloning steps affected the overall expression of previously integrated retroviral components. Therefore, viral titers differences obtained between 293 FLEX and dFLEX are strictly due to the envelope expression.

dFLEX cells were subsequently challenged to produce retroviral particles pseudotyped with VSV-G envelope protein. This envelope is of high industrial interest due to its broad range tropism as well as enhanced stability nonetheless is associated with marked cytotoxicity which prevents long-term cell cultures. The introduction of inducible expression systems minimized VSV-G-associated cytotoxicity and enabled the development of VSV-G pseudotyped retroviral vectors. Here, dFLEX cells producing VSV-G pseudotypes under the control of an inducible gene expression system, using an autoregulated module, extended the advantages of dFLEX multiplexing system also to express cytotoxic proteins and validated the tagged chromosomal locus to support regulated gene expression systems.

In summary, herein we report the development of a novel cell line, dFLEX cells, for extended and tailored production of diverse recombinant retroviral products. dFLEX cells show flexibility of envelope expression while maintaining high production titers of infectious particles.

## Electronic supplementary material

Below is the link to the electronic supplementary material.

Supplementary material 1 (DOCX 35 kb)

## References

[CR1] Anderson RP, Voziyanova E, Voziyanov Y (2012). Flp and Cre expressed from Flp-2A-Cre and Flp-IRES-Cre transcription units mediate the highest level of dual recombinase-mediated cassette exchange. Nucleic Acids Res.

[CR2] Carrondo MJT, Merten OW, Haury M, Alves PM, Coroadinha AS (2008). Impact of retroviral vector components stoichiometry on packaging cell lines: effects on productivity and vector quality. Hum Gene Ther.

[CR3] Cone RD, Mulligan RC (1984). High-efficiency gene transfer into mammalian cells: generation of helper-free recombinant retrovirus with broad mammalian host range. Proc Natl Acad Sci USA.

[CR4] Coroadinha AS, Alves PM, Santos SS, Cruz PE, Merten O-W, Carrondo MJT (2006). Retrovirus producer cell line metabolism: implications on viral productivity. Appl Microbiol Biotechnol.

[CR5] Coroadinha AS, Ribeiro J, Roldão A, Cruz PE, Alves PM, Merten O-W, Carrondo MJT (2006). Effect of medium sugar source on the production of retroviral vectors for gene therapy. Biotechnol Bioeng.

[CR6] Coroadinha AS, Schucht R, Gama-Norton L, Wirth D, Hauser H, Carrondo MJT (2006). The use of recombinase mediated cassette exchange in retroviral vector producer cell lines: predictability and efficiency by transgene exchange. J Biotechnol.

[CR7] Coroadinha AS, Silva AC, Pires E, Coelho A, Alves PM, Carrondo MJT (2006). Effect of osmotic pressure on the production of retroviral vectors: enhancement in vector stability. Biotechnol Bioeng.

[CR8] Danos O, Mulligan RC (1988). Safe and efficient generation of recombinant retroviruses with amphotropic and ecotropic host ranges. Proc Natl Acad Sci USA.

[CR9] Debyser Z, Christ F, De Rijck J, Gijsbers R (2015). Host factors for retroviral integration site selection. Trends Biochem Sci.

[CR10] Kameyama Y, Kawabe Y, Ito A, Kamihira M (2010). An accumulative site-specific gene integration system using Cre recombinase-mediated cassette exchange. Biotechnol Bioeng.

[CR11] Maetzig T, Galla M, Baum C, Schambach A (2011). Gammaretroviral vectors: biology, technology and application. Viruses.

[CR12] Miller AD (1990). Retrovirus packaging cells. Hum Gene Ther.

[CR13] Rodrigues AF, Formas-Oliveira AS, Bandeira VS, Alves PM, Hu WS, Coroadinha AS (2013). Metabolic pathways recruited in the production of a recombinant enveloped virus: mining targets for process and cell engineering. Metab Eng.

[CR14] Schambach A, Mueller D, Galla M, Verstegen MMA, Wagemaker G, Loew R, Baum C, Bohne J (2006). Overcoming promoter competition in packaging cells improves production of self-inactivating retroviral vectors. Gene Ther.

[CR15] Schucht R, Coroadinha AS, Zanta-Boussif MA, Verhoeyen E, Carrondo MJT, Hauser H, Wirth D (2006). A new generation of retroviral producer cells: predictable and stable virus production by Flp-mediated site-specific integration of retroviral vectors. Mol Ther.

[CR16] Soares HR, Castro R, Tomás HA, Rodrigues AF, Gomes-Alves P, Bellier B, Klatzmann D, Carrondo MJT, Alves PM, Coroadinha AS (2016). Tetraspanins displayed in retrovirus-derived virus-like particles and their immunogenicity. Vaccine.

[CR17] Turan S, Kuehle J, Schambach A, Baum C, Bode J (2010). Multiplexing RMCE: versatile extensions of the Flp-recombinase-mediated cassette-exchange technology. J Mol Biol.

[CR18] Turan S, Galla M, Ernst E, Qiao J, Voelkel C, Schiedlmeier B, Zehe C, Bode J (2011). Recombinase-mediated cassette exchange (RMCE): traditional concepts and current challenges. J Mol Biol.

[CR19] Turan S, Zehe C, Kuehle J, Qiao J, Bode J (2013). Recombinase-mediated cassette exchange (RMCE) - a rapidly-expanding toolbox for targeted genomic modifications. Gene.

[CR20] Turan S, Qiao J, Madden S, Benham C, Kotz M, Schambach A, Bode J (2014). Expanding Flp-RMCE options: the potential of recombinase mediated twin-site targeting (RMTT). Gene.

