# Supplementary material for: Flexible pseudotyping of retrovirus using recombinase-mediated cassette exchange
Source: Biotechnol Lett. 2018 Jan 20;40(4):633–9. doi: 10.1007/s10529-018-2515-6 (PMC5862940; doi:10.1007/s10529-018-2515-6)
Supplement: Supplementary file 1 — Supplementary material 1 (DOCX 35 kb) [file 10529_2018_2515_MOESM1_ESM.docx]

**Supplementary Information**

**Supplementary Materials and Methods**

**Cell Lines and Culture Media**

293#3 gp11 and 293#3 gp22 (Carrondo et al., 2008; Coroadinha et al., 2006) are two human embryonic kidney (HEK 293, ATCC CRL-1573) derived cell lines continuously producing non-pseudotyped murine leukemia virus-based vectors harboring a *lacZ* reporter gene flanked by Flp/*FRT* recombination sites as described in Coroadinha et al. (2006c). Flex#18 cells derive from 293#3 gp22 by stable transfection of GaLV envelope expression plasmid (Carrondo et al., 2008). HEK293T (ATCC CRL-11268) cells were used for transient transfections and RD cells (ATCC CCL-136), were used as target cells to titrate infectious retroviral particles. All cells were maintained in Dulbecco’s modified Eagle’s medium, DMEM, (Gibco) supplemented with 10 % (v / v) Foetal Bovine Serum (FBS) (Gibco) at 37 ºC inside an incubator with an humidified atmosphere of 5 % CO2 in air.

**Plasmids**

The pTagLoxPmcs plasmid used as backbone for the cell tagging vector was synthesized by GeneArt (Thermo Fisher Scientific). Synthesized plasmid contains Lox2272/66 and LoxP/71 (Kolb, 2001; Lee and Saito, 1998) sites for Cre recombination and a promoterless puromycin resistance gene. The CMV-GFPZeo-polyA sequence was amplified from pselect–GFPZeo-LacZ (InvivoGen) by PCR and cloned into the pTagLoxPmcs vector generating the final plasmid pTagLoxPGFP-zeo. Targeting plasmid backbone, pTarLoxmcs, was synthesized by GenScript (Piscataway, NJ U.S.A.) containing a Lox2272/71 and a Lox P/66 Cre-recognition sites, a CMV promoter and a rabbit β-globin intron followed by a multiple cloning site (MCS). pZeoCre was used to express Cre recombinase (Fernandes et al., 2013). The composite hybrid promoter SV40 enhancer, ferritin heavy chain and mouse elongation factor (SV40/FerH/mEF1α) was amplified from pMonoZeoMCS (InvivoGen) and cloned into the multiple cloning site of pTarLoxmcs generating the final plasmid pTarLoxFerH. MLV 4070A and GaLV envelopes were cloned upstream the composite FerH promoter by PCR amplification and plasmid restriction generating the final plasmids pTarLox4070A and pTarLoxGaLV respectively. MLV4070A and GalV10A1 derived from pENVAhis (Schucht et al., 2006) and pGaLV (Coroadinha et al., 2006), respectively. The inducible tetracycline promoter was isolated from pRITA (May et al., 2004) (kindly provided by Dr. Dagmar Wirth, Helmholtz Centre for Infection Research, Germany) and subcloned into pTarLoxFerH, generating the final plasmid pTarLoxTetFerH. VSV-G envelope gene was amplified from pMD2.G plasmid (Addgene plasmid 12259) kindly provided by D. Trono through the Addgene plasmid repository (Cambridge, MA U.S.A.), and cloned downstream of the inducible promoter and upstream of the FerH promoter generating the final plasmid pTarLoxVSVG. All cloning reactions described above were conducted using In-Fusion HD Cloning system (Takara, Mountain View, CA U.S.A.) combining different PCR amplification, endonuclease restriction and primer sets strategies for accurate molecular cloning. All plasmids were sanger-sequenced before use and DNA sequences of plasmids and primers are available upon request.

**Production of pseudotyped particles**

Retroviral particles production was performed as described before (Coroadinha et al., 2006), briefly cells were seeded at 5x10^4^ cells cm^-2^ in T25 tissue-culture flasks (Sarstedt, Nümbrecht, Germany) and cultured until 80 % confluence. The cell culture medium was then replaced with new DMEM 10 % (v/v) FBS, plain or supplemented with 2 µg ml^-1^ doxycycline in the case of VSV-G pseudotyped particles. After 24 h production, cell culture containing viral particles was harvested, filtered with a 0.45 μm pore membrane and diluted before titration on target cells.

**PCR and RT-PCR**

DNA was isolated from 5x10^6^ cells using the QIAamp® DNA Mini and Blood Mini Kit (Qiagen). 250 ng of genomic DNA was mixed with specific primers annealing with sequences flanking LoxP/66 recombination site and with GoTaq® DNA Polymerase (Promega). The primers used for eGFP amplification were: Fw1-5’ATTGACGCAAATGGGCGGT3’ and Rev1-5’GTGTTCTGCTGGTAATGGTC3’; and for targeted versus random integration Fw2-5’TGCCTTCGATTGCCGTTCAG3’ and Rev2-5’TCAGTGAGCGAGGAAGCGGA3’. To estimate the relative expression of lacZ and MLV gag-pol genes total RNA was extracted using RNeasy Mini Kit (Qiagen) and eluted in 100 µL of nuclease-free water (Qiagen) before storage at -85 ºC. The reverse transcription of total RNA and qRT-PCR was performed as described before (Rodrigues et al., 2013, 2012). Primer used for lacZ amplification were Fw–5’ACTATCCCGACCGCCTTACT3’ and Rev-5’TAGCGGCTGATGTTGAACTG3’; for GagPol Fw–5’GTCCACTATCGCCAGTTGCT3’ and Rev – 5’CTGGGTCCTCAGGGTCATAA3’.

**Western Blotting**

Cell lysates were prepared by adding 100 µL of M-PER extraction buffer (Thermo Scientific) to 1x10^6^ cells and mix. 16 µg of cell lysates were separated in a 4–12 % (w/v) acrylamide NuPAGE gradient pre-cast gel (LifeTechnologies). Samples were resolved for 45 min at a constant voltage of 180 V and transferred into a PVDF membrane using a Trans-Blot® Turbo™ Transfer System (BioRad, California, USA). After transfering, membranes were blocked with 4 % (w/v) skimmed milk (Merck Millipore) and incubated with the respective primary antibody: rabbit anti-VSV-G (Sigma-Aldrich), mouse β-actin (Abcam, Cambridge, United Kingdom). Detection was performed with the respective anti-rabbit and anti-mouse secondary antibodies conjugated to horseradish peroxidase (GE Healthcare) and developed using the ECL Detection Reagent (GE Healthcare).

**Supplementary References**

Carrondo, M.J.T., Merten, O.-W., Haury, M., Alves, P.M., Coroadinha, A.S., 2008. Impact of retroviral vector components stoichiometry on packaging cell lines: effects on productivity and vector quality. Hum. Gene Ther. 19, 199–210. doi:10.1089/hum.2007.0101

Coroadinha, A.S., Schucht, R., Gama-Norton, L., Wirth, D., Hauser, H., Carrondo, M.J.T., 2006. The use of recombinase mediated cassette exchange in retroviral vector producer cell lines: predictability and efficiency by transgene exchange. J. Biotechnol. 124, 457–68. doi:10.1016/j.jbiotec.2006.01.037

Fernandes, P., Santiago, V.M., Rodrigues, A.F., Tomás, H., Kremer, E.J., Alves, P.M., Coroadinha, A.S., 2013. Impact of E1 and Cre on Adenovirus Vector Amplification: Developing MDCK CAV-2-E1 and E1-Cre Transcomplementing Cell Lines. PLoS One 8. doi:10.1371/journal.pone.0060342

Kolb, A.F., 2001. Selection-marker-free modification of the murine beta-casein gene using a lox2272 [correction of lox2722] site. Anal. Biochem. 290, 260–71. doi:10.1006/abio.2000.4984

Lee, G., Saito, I., 1998. Role of nucleotide sequences of loxP spacer region in Cre-mediated recombination. Gene 216, 55–65. doi:10.1016/S0378-1119(98)00325-4

May, T., Hauser, H., Wirth, D., 2004. Transcriptional control of SV40 T-antigen expression allows a complete reversion of immortalization. Nucleic Acids Res. 32, 5529–5538. doi:10.1093/nar/gkh887

Rodrigues, A.F., Amaral, A.I., Veríssimo, V., Alves, P.M., Coroadinha, a S., 2012. Adaptation of retrovirus producer cells to serum deprivation: Implications in lipid biosynthesis and vector production. Biotechnol. Bioeng. 109, 1269–79. doi:10.1002/bit.24410

Rodrigues, A.F., Formas-Oliveira, A.S., Bandeira, V.S., Alves, P.M., Hu, W.S., Coroadinha, a S., 2013. Metabolic pathways recruited in the production of a recombinant enveloped virus: mining targets for process and cell engineering. Metab. Eng. 20, 131–45. doi:10.1016/j.ymben.2013.10.001

Schucht, R., Coroadinha, a. S., Zanta-Boussif, M. a., Verhoeyen, E., Carrondo, M.J.T., Hauser, H., Wirth, D., 2006. A New Generation of Retroviral Producer Cells: Predictable and Stable Virus Production by Flp-Mediated Site-Specific Integration of Retroviral Vectors. Mol. Ther. 14, 285–292. doi:10.1016/j.ymthe.2005.12.003
